# Association of plasma free amino acids with hyperuricemia in relation to diabetes mellitus, dyslipidemia, hypertension and metabolic syndrome

**DOI:** 10.1038/s41598-017-17710-6

**Published:** 2017-12-15

**Authors:** MH Mahbub, Natsu Yamaguchi, Hidekazu Takahashi, Ryosuke Hase, Yasutaka Ishimaru, Hiroshi Sunagawa, Hiroki Amano, Mikiko Kobayashi-Miura, Hideyuki Kanda, Yasuyuki Fujita, Hiroshi Yamamoto, Mai Yamamoto, Shinya Kikuchi, Atsuko Ikeda, Naoko Kageyama, Mina Nakamura, Tsuyoshi Tanabe

**Affiliations:** 1 Department of Public Health and Preventive Medicine, Graduate School of Medicine, Yamaguchi University, Ube, Japan; 2 Division of Health Administration and Promotion, Graduate School of Medicine, Tottori University, Yonago, Japan; 3 Department of Biochemistry, Faculty of Medicine, Shimane University, Izumo, Japan; 4 Department of Environmental Medicine and Public Health, Faculty of Medicine, Shimane University, Izumo, Japan; 5 Research Institute for Bioscience Products & Fine Chemicals, Ajinomoto Co., Inc., Kawasaki, Japan; 6 Institute for Innovation, Ajinomoto Co., Inc., Kawasaki, Japan

## Abstract

Previous studies demonstrated independent contributions of plasma free amino acids (PFAAs) and high uric acid (UA) concentrations to increased risks of lifestyle-related diseases (LSRDs), but the important associations between these factors and LSRDs remain unknown. We quantified PFAAs and UA amongst Japanese subjects without LSRDs (no-LSRD, n = 2805), and with diabetes mellitus (DM, n = 415), dyslipidemia (n = 3207), hypertension (n = 2736) and metabolic syndrome (MetS, n = 717). The concentrations of most amino acids differed significantly between the subjects with and without hyperuricemia (HU) and also between the no-LSRD and LSRD groups (p < 0.05 to 0.001). After adjustment, the logistic regression analyses revealed that lysine in DM, alanine, proline and tyrosine in dyslipidemia, histidine, lysine and ornithine in hypertension, and lysine and tyrosine in MetS demonstrated significant positive associations with HU among the patients with LSRDs only (p < 0.05 to 0.005). By contrast, arginine, asparagine and threonine showed significant inverse associations with HU in the no-LSRD group only (p < 0.05 to 0.01). For the first time, we provide evidence for distinct patterns of association between PFAAs and HU in LSRDs, and postulate the possibility of interplay between PFAAs and UA in their pathophysiology.

## Introduction

In the human body, plasma amino acid imbalances occur in various diseases, and such imbalances may exert specific negative effects on various physiological processes and organ functions^1^. Several recent studies have demonstrated a strong correlation between alterations in the concentrations of various plasma free amino acids (PFAAs) and the development of lifestyle-related diseases (LSRDs), including diabetes mellitus (DM), dyslipidemia, hypertension and metabolic syndrome (MetS)^2–5^. These reports suggested that the changes in amino acid metabolism play important roles in the pathogenesis of LSRDs. According to the findings of existing studies, those analytes can be predictive of the development of LSRDs, serve as effective biomarkers for their detection and be highly responsive to therapeutic interventions^4–8^. Conversely, in recent years, the prevalence of hyperuricemia (HU) has been increasing worldwide^9^. A growing number of published studies reported a strong association of elevated uric acid (UA) levels in the blood with LSRDs^10–15^. Several epidemiological studies described HU as an independent risk factor and predictor for the development and progression of various LSRDs^12,16–19^. Considering the significant association of HU with MetS and all its individual components, it has been suggested that the level of UA could be included as an independent component in the definition of MetS^20^. From the findings of the above-mentioned old and recent studies, it is understandable that both PFAAs and UA are independently and significantly associated with LSRDs.

However, some published reports exhibited a clear association between blood UA and purine-rich foods, given that the former is the end product of purine metabolism^21,22^. Certain amino acids participate in the biosynthesis of purine and the subsequent formation of UA^23^. In this context, we recently showed that plasma levels of several branched chain amino acids (BCAAs) and aromatic amino acids (AAAs) had significant positive associations with gout, a disease with elevated levels of UA in the blood^24^.

Taken together, the existing scientific evidence indicates that PFAAs may be linked to plasma UA, and the abnormalities in LSRDs may be the result of combined effects of both amino acids and UA. Understanding the association between PFAAs and UA in LSRDs is of utmost importance because it might help to better apprehend the disease pathophysiology and aid in the prevention and/or early detection and treatment of LSRDs, and thereby alleviate the outcomes and burden associated with the latter. However, any relationships between PFAAs and UA in LSRDs remain unclear as no studies, to the best of our knowledge, have hitherto investigated it in the general population.

Therefore, the purpose of this cross-sectional study was to examine the differences in the concentrations of PFAAs amongst subjects with and without HU divided into groups without LSRDs (no-LSRD) and with LSRDs (DM, dyslipidemia, hypertension and MetS). Furthermore, we attempted to explore and clarify the association of PFAAs with HU in LSRDs.

## Results

### Demographic and clinical characteristics of the study subjects

**Figure 1:**
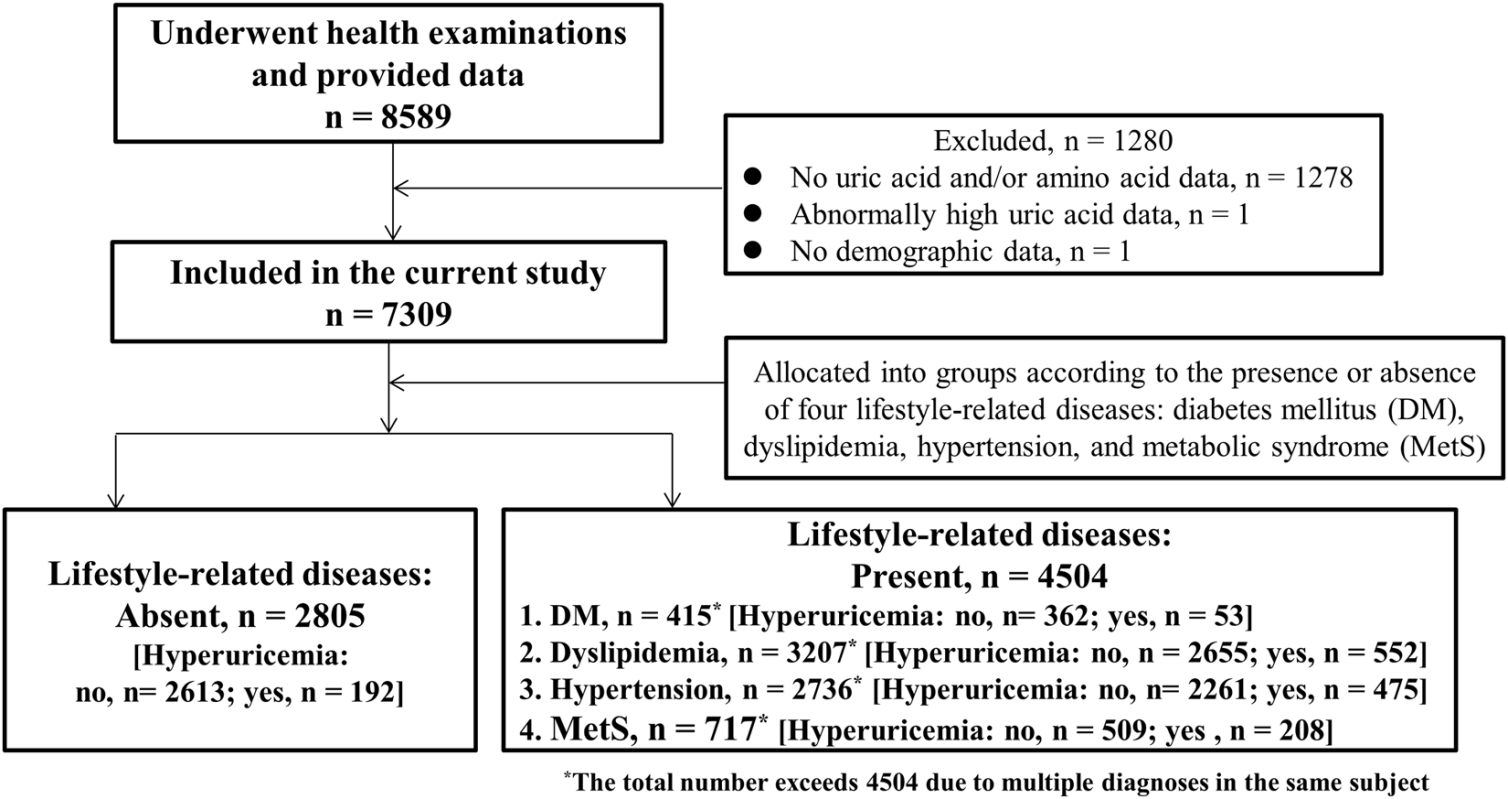
Flowchart of study participants.

A total of 4504 subjects with LSRDs and 2805 subjects (1192 men and 1613 women) without LSRDs were included in this study (Fig. 1). Amongst the patients with LSRDs, 415 (265 men and 150 women) had DM, 3207 (1617 men and 1590 women) had dyslipidemia, 2736 (1473 men and 1263 women) had hypertension, and 717 (532 men and 185 women) had MetS. Compared to the no-LSRD group, patients with LSRDs exhibited significantly higher levels of UA (median and interquartile range/IQR values were 4.6 and 1.8 mg/dl, 5.1 and 1.9 mg/dl, 5.2 and 2.0 mg/dl, 5.3 and 2.0 mg/dl, and 5.9 and 2.0 mg/dl for no-LSRD, DM, dyslipidemia, hypertension, and MetS groups, respectively) (Kruskal-Wallis test, p < 0.001). Furthermore, when compared to the no-LSRD group, patients with LSRDs exhibited a higher prevalence of HU (6.8% for no-LSRD versus 12.9 to 29.0% for LSRDs). The prevalence of HU amongst men (5.4 to 23%) was much higher than that amongst women (1.4 to 6%) in each of the five groups of subjects: no-LSRD, men 5.4% versus women 1.4% (χ^2^ test, p < 0.001); DM, men 8% versus women 4.8% (χ^2^ test, p = 0.074); dyslipidemia, men 12.4% versus women 4.8% (χ^2^ test, p < 0.001); hypertension, men 12.4% versus women 4.9% (χ^2^ test, p < 0.001); and MetS, men 23.0% versus women 6.0% (χ^2^ test, p < 0.001).

The demographic and clinical characteristics of the study populations have been presented and compared between the subjects with and without HU, and the groups with and without LSRDs in Table 1. In each of the 5 study groups, except in DM, the majority of the demographic and clinical variables differed significantly between the subjects with and without HU (Mann-Whitney U-test, p < 0.05 to 0.001). Furthermore, for all those variables, the differences between the subjects with and without LSRDs were highly significant (Kruskal-Wallis test, p < 0.001). Compared to the patients with LSRDs, the subjects without LSRDs were younger and had a lower BMI and waist circumference. Conversely, all groups of patients with LSRDs exhibited significantly higher values for systolic blood pressure (SBP), diastolic blood pressure (DBP), low-density lipoprotein cholesterol (LDLC), triglyceride (TG), fasting plasma glucose (FPG), and hemoglobin A1c (HbA1c); by contrast, the level of high-density lipoprotein cholesterol (HDLC) was lower in the patients with LSRDs. The differences between the no-LSRD group and each of the other four groups of patients with LSRDs remained significant for all the demographic and clinical variables after adjustments for multiple comparisons (Bonferroni-corrected Mann-Whitney U-test, p < 0.05 to 0.001) except only for LDLC in HU for DM (p > 0.05).

**Table 1:** Demographic and clinical characteristics of the study subjects without lifestyle-related diseases (no-LSRD) and with diabetes mellitus (DM), dyslipidemia, hypertension and metabolic syndrome (MetS). Differences in the number of subjects (n) under different groups corresponding to each variable are due to varying missing data.

Variable	HU	No-LSRD				DM					Dyslipidemia					Hypertension					MetS					p3
		n	Median	IQR	p1	n	Median	IQR	p1	p2	n	Median	IQR	p1	p2	n	Median	IQR	p1	p2	n	Median	IQR	p1	p2	
Age(Years)	No	2613	44.0	26.5	0.223	362	65.5	15.3	0.407	<0.001	2655	62.0	19.0	<0.001	<0.001	2261	66.0	16.0	<0.001	<0.001	509	61.0	17.0	0.001	<0.001	<0.001
	Yes	192	41.5	25.0		53	68.0	14.5		<0.001	552	56.5	26.0		<0.001	475	63.0	20.0		<0.001	208	58.0	20.0		<0.001	<0.001
BMI(kg/m^2^)	No	2613	21.1	3.6	<0.001	362	23.8	4.3	<0.001	<0.001	2655	23.1	4.0	<0.001	<0.001	2261	23.3	4.1	<0.001	<0.001	509	26.2	3.4	0.001	<0.001	<0.001
	Yes	192	23.1	3.4		53	26.6	5.4		<0.001	552	24.5	4.3		<0.001	475	24.5	4.5		<0.001	208	27.0	4.2		<0.001	<0.001
FPG(mg/dL)	No	2424	90.0	10.0	0.002	337	130.0	46.5	0.436	<0.001	2388	95.0	14.0	0.002	<0.001	2006	98.0	14.0	0.198	<0.001	466	107.0	26.3	<0.001	<0.001	<0.001
	Yes	181	93.0	10.0		50	132.0	43.3		<0.001	492	97.0	13.0		<0.001	419	99.0	13.0		<0.001	191	101.0	18.0		<0.001	<0.001
HbA1c(%)	No	2613	5.5	0.5	0.174	362	7.0	1.2	0.327	<0.001	2655	5.7	0.5	0.057	<0.001	2261	5.8	0.6	0.032	<0.001	509	6.0	1.0	<0.001	<0.001	<0.001
	Yes	192	5.5	0.5		53	6.9	1.1		<0.001	552	5.7	0.5		<0.001	475	5.7	0.5		<0.001	208	5.9	0.8		<0.001	<0.001
HDLC(mg/dL)	No	2613	68.0	21.0	<0.001	362	54.0	19.3	0.268	<0.001	2655	58.0	22.0	<0.001	<0.001	2261	61.0	21.0	<0.001	<0.001	509	53.0	18.5	0.085	<0.001	<0.001
	Yes	192	61.0	22.0		53	52.0	20.0		0.003	552	52.5	19.0		<0.001	475	56.0	22.0		0.002	208	50.0	19.0		<0.001	<0.001
LDLC(mg/dL)	No	2613	107.0	31.0	0.135	362	119.0	36.3	0.878	<0.001	2655	142.0	42.0	0.199	<0.001	2261	119.0	38.0	0.958	<0.001	509	123.0	38.0	0.598	<0.001	<0.001
	Yes	192	110.0	31.0		53	120.0	48.0		ns	552	140.0	48.0		<0.001	475	119.0	45.0		<0.001	208	121.0	41.8		<0.001	<0.001
TG(mg/dL)	No	2613	63.0	36.0	<0.001	362	100.0	75.0	0.001	<0.001	2655	106.0	77.0	<0.001	<0.001	2261	92.0	56.0	<0.001	<0.001	509	147.0	94.0	<0.001	<0.001	<0.001
	Yes	192	84.5	40.5		53	123.0	90.5		<0.001	552	157.0	104.0		<0.001	475	118.0	92.0		<0.001	208	168.5	111.8		<0.001	<0.001
SBP(mmHg)	No	2613	118.0	16.0	<0.001	362	134.0	24.0	0.045	<0.001	2655	129.0	21.0	<0.001	<0.001	2261	141.0	18.0	0.404	<0.001	509	138.0	18.0	0.913	<0.001	<0.001
	Yes	192	122.5	14.0		53	138.0	17.5		<0.001	552	132.0	21.0		<0.001	475	140.0	18.0		<0.001	208	138.0	18.0		<0.001	<0.001
DBP(mmHg)	No	2613	73.0	12.0	<0.001	362	79.0	17.0	0.581	<0.001	2655	79.0	14.0	<0.001	<0.001	2261	85.0	14.0	<0.001	<0.001	509	86.0	12.0	0.083	<0.001	<0.001
	Yes	192	77.0	12.0		53	81.0	14.5		0.046	552	82.5	15.0		<0.001	475	90.0	15.0		<0.001	208	87.5	14.0		<0.001	<0.001
Waist(cm)	No	2530	75.5	11.0	<0.001	325	85.5	11.0	<0.001	<0.001	2467	83.5	10.5	<0.001	<0.001	1946	84.0	10.8	<0.001	<0.001	509	92.0	8.5	0.034	<0.001	<0.001
	Yes	188	82.0	10.4		49	91.0	12.5		<0.001	531	87.0	11.0		<0.001	437	87.5	12.3		<0.001	208	93.0	9.0		<0.001	<0.001

p1 indicates the p-values for the two-tailed Mann-Whitney U-test for 2-independent samples (shown under each group of subjects); p2 indicates the p-values for the two-tailed Mann-Whitney U-test for 2-independent samples with adjustments by Bonferroni corrections for multiple comparisons with the corresponding values of no-LSRD group (shown under DM, dyslipidemia, hypertension and MetS); and p3 indicates the p-values for the two-tailed Kruskal-Wallis test for k-independent samples (shown in the last column). ns, not significant after adjustment for multiple comparisons.

The percentages of subjects taking medications for the respective diseases in the four groups of patients with LSRDs were as follows: DM- no HU, 273/357 or 76.5% (missing, n = 5), yes HU, 41/52 or 78.8% (missing, n = 1); dyslipidemia- no HU, 833/2559 or 32.6% (missing, n = 96), yes HU, 135/534 or 25.3% (missing, n = 18); hypertension- no HU, 1322/2232 or 59.2% (missing, n = 29), yes HU, 260/465 or 55.9% (missing, n = 10); and MetS- no HU, 318/503 or 63.2% (missing, n = 6), yes HU, 119/204 or 58.3% (missing, n = 4). With respect to the use of medications, no significant differences could be revealed by the χ^2^ test between the patients with and without HU in DM (p = 0.705), hypertension (p = 0.187) and MetS (p = 0.226) except in dyslipidemia (p = 0.001).

### Correlation between PFAAs and UA

**Figure 2:**
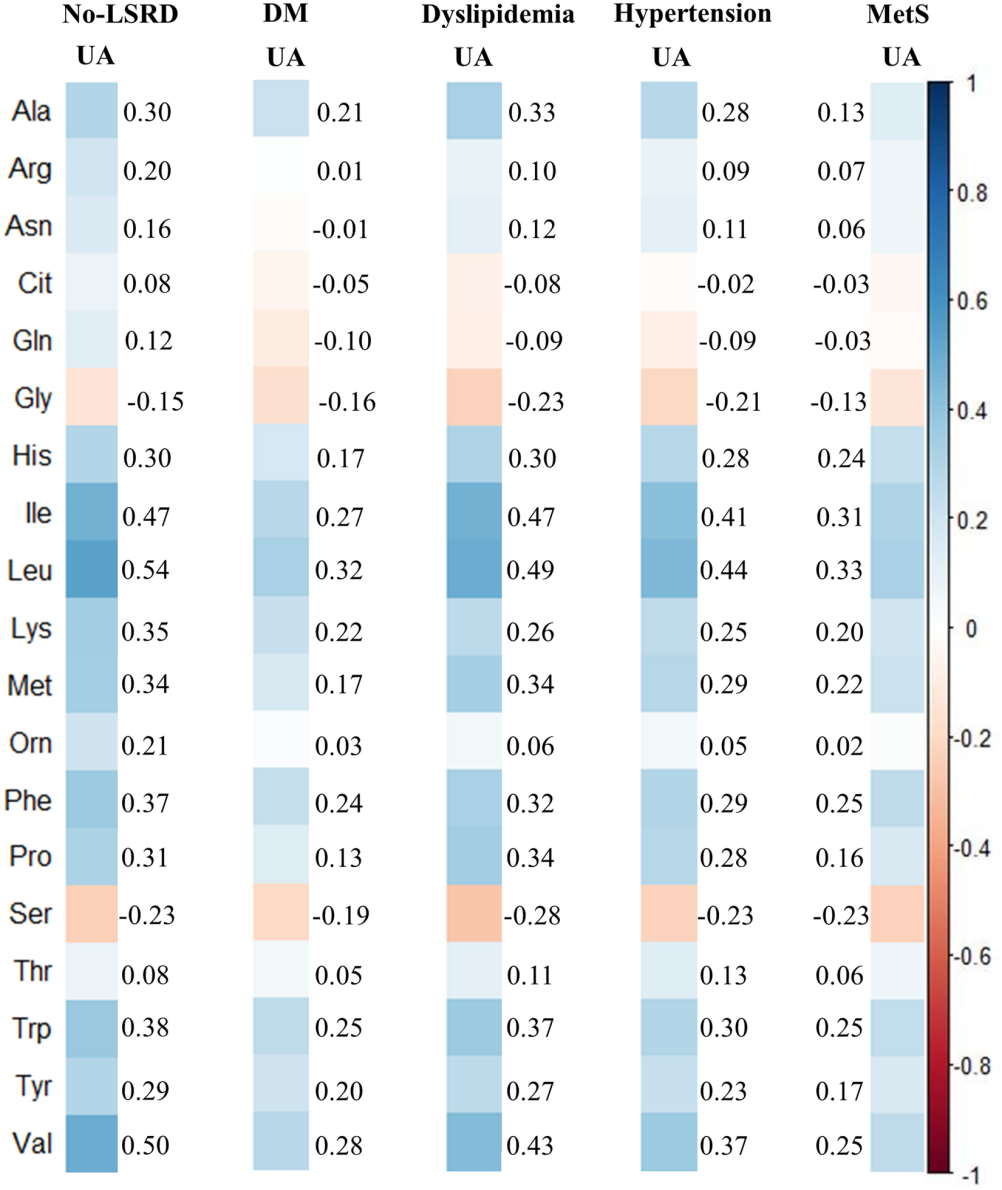
Color map showing the correlation coefficients derived by Spearman rank correlation analysis between the concentrations of plasma free amino acids (PFAAs, μmol/L) and uric acid (UA, mg/dL) for subjects without lifestyle-related diseases (no-LSRD) and with diabetes mellitus (DM), dyslipidemia, hypertension and metabolic syndrome (MetS). Blue: positive correlation; brown: negative correlation.

At first, we examined the relationship between PFAAs measured in this study and UA with a correlation analysis in the no-LSRD group and the LSRD groups (Fig. 2). As evident in the figure, the concentrations of PFAAs were correlated to UA in both the no-LSRD and LSRD groups, and the correlations demonstrated similar trends in both groups. Overall, glycine (Gly) and serine (Ser) showed negative correlations (Spearman’s rank correlation, r = −0.13 to −0.28; p < 0.005 to 0.001), and alanine (Ala), histidine (His), isoleucine (Ile), leucine (Leu), lysine (Lys), methionine (Met), phenylalanine (Phe), proline (Pro), tryptophan (Trp), tyrosine (Tyr), and valine (Val) showed positive correlations (Spearman’s rank correlation, r = 0.13 to 0.54; p < 0.005 to 0.001) with UA that achieved relatively higher values in most of the investigated groups.

### Differences in amino acid concentrations

As shown in Table 2, the concentrations of most of the amino acids differed significantly between the subjects with and without HU (Mann-Whitney U-test, p < 0.05 to 0.001) in all groups; the exception was DM, in which 7 out of 19 amino acids showed such significant differences. Compared to the subjects without HU, the analyses revealed a significant increase in the concentrations of BCAAs (Ile, Leu and Val) in all groups of subjects with HU (Mann-Whitney U-test, p < 0.05 to 0.001) (Table 2). Such a significant increase in those subjects was also observed for the AAAs (Phe, Trp and Tyr; Mann-Whitney U-test, p < 0.05 to 0.001), with exceptions for Trp and Tyr in DM. Amongst the other amino acids, the hyperuricemic subjects showed a significant increase in most of the investigated groups in the concentrations of Ala (except DM and MetS), His (except DM), Lys, and Met (except DM) (Mann-Whitney U-test, p < 0.05 to 0.001). Conversely, a significantly lower concentration of Ser was observed amongst the subjects with HU in all groups (Mann-Whitney U-test, p < 0.01 to 0.001). Moreover, reduced concentrations of glutamine (Gln) (except in no-LSRD and MetS) and Gly (except DM) were observed in those subjects (Mann-Whitney U-test, p < 0.05 to 0.001).

**Table 2:** Plasma free amino acid concentrations (μmol/L) in the study subjects without lifestyle-related diseases (no-LSRD) and with diabetes mellitus (DM), dyslipidemia, hypertension and metabolic syndrome (MetS).

Amino acid	HU	No-LSRD			DM				Dyslipidemia				Hypertension				MetS				p3
		(HU: no, 2613; yes, 192)			(HU: no, 362; yes, 53)				(HU: no, 2655; yes, 552)				(HU: no, 2261; yes, 475)				(HU: no, 509; yes, 208)				
		Median	IQR	p1	Median	IQR	p1	p2	Median	IQR	p1	p2	Median	IQR	p1	p2	Median	IQR	p1	p2	
Ala	No	308.3	93.7	<0.001	373.9	121.1	0.076	<0.001	341.5	110.5	<0.001	<0.001	341.7	107.6	<0.001	<0.001	392.7	103.7	0.440	<0.001	<0.001
	Yes	342.2	103.0		408.8	117.5		<0.001	383.0	101.5		<0.001	372.5	99.1		<0.001	396.8	106.6		<0.001	<0.001
Arg	No	91.7	24.1	0.274	98.8	27.5	0.167	<0.001	95.7	22.4	0.292	<0.001	95.9	22.9	0.483	<0.001	97.6	24.5	0.697	<0.001	<0.001
	Yes	93.3	27.7		91.2	32.3		ns	97.4	21.7		0.026	97.1	21.9		0.042	98.0	22.3		0.026	0.038
Asn	No	45.1	9.2	0.770	46.2	10.0	0.160	0.035	44.8	8.4	0.213	ns	45.0	9.0	0.308	ns	45.2	8.9	0.663	ns	0.007
	Yes	45.0	8.0		43.8	9.6		ns	45.1	8.8		ns	45.1	8.2		ns	45.1	8.1		ns	0.922
Cit	No	28.0	8.8	0.357	31.9	11.7	0.530	<0.001	30.5	9.0	0.009	<0.001	31.3	9.9	0.751	<0.001	29.3	9.2	0.990	<0.001	<0.001
	Yes	28.7	9.6		33.9	14.9		0.034	29.2	10.4		ns	30.8	11.0		<0.001	29.3	11.1		ns	<0.001
Gln	No	584.7	89.3	0.324	599.7	101.1	0.037	0.001	600.3	84.8	<0.001	<0.001	599.5	89.4	<0.001	<0.001	591.6	94.0	0.429	ns	<0.001
	Yes	576.1	94.0		577.4	101.1		ns	585.4	89.8		ns	583.9	92.2		ns	589.3	101.0		ns	0.803
Gly	No	219.8	62.0	<0.001	197.1	53.3	0.130	<0.001	207.1	60.7	<0.001	<0.001	204.5	61.1	<0.001	<0.001	192.2	47.6	0.019	<0.001	<0.001
	Yes	202.4	53.5		191.2	52.4		ns	191.1	44.0		ns	192.5	51.2		ns	186.8	43.4		0.001	0.007
His	No	77.8	12.2	<0.001	80.2	12.1	0.271	<0.001	79.0	12.0	<0.001	<0.001	78.6	12.0	<0.001	0.037	80.5	13.7	<0.001	<0.001	<0.001
	Yes	80.7	12.3		81.6	14.2		ns	83.1	13.0		ns	82.3	12.4		ns	84.1	14.1		0.031	0.035
Ile	No	50.8	15.5	<0.001	63.2	19.9	0.040	<0.001	56.4	18.1	<0.001	<0.001	56.1	18.1	<0.001	<0.001	64.5	19.8	<0.001	<0.001	<0.001
	Yes	60.1	15.5		68.1	22.0		0.009	66.4	19.7		<0.001	64.2	19.2		ns	69.9	18.8		<0.001	<0.001
Leu	No	101.3	28.4	<0.001	122.9	32.9	0.003	<0.001	111.8	32.7	<0.001	<0.001	110.6	32.1	<0.001	<0.001	126.0	32.5	<0.001	<0.001	<0.001
	Yes	120.9	27.0		133.7	32.4		0.003	128.9	32.6		0.001	124.3	31.0		ns	134.8	31.2		<0.001	<0.001
Lys	No	174.7	43.4	<0.001	193.1	41.7	0.025	<0.001	186.6	40.2	<0.001	<0.001	184.9	40.1	<0.001	<0.001	196.1	39.5	0.003	<0.001	<0.001
	Yes	186.7	46.0		201.4	42.9		0.002	197.7	38.3		0.001	196.1	41.8		0.019	202.3	36.7		<0.001	<0.001
Met	No	24.0	6.1	<0.001	26.6	6.4	0.697	<0.001	24.5	6.1	<0.001	<0.001	24.8	6.2	<0.001	<0.001	26.7	6.0	0.015	<0.001	<0.001
	Yes	25.9	7.0		27.2	7.8		ns	26.3	6.2		ns	26.2	6.5		ns	27.4	5.7		0.001	0.002
Orn	No	43.8	13.1	0.004	51.7	16.0	0.422	<0.001	48.6	13.0	0.120	<0.001	49.6	12.9	0.083	<0.001	50.2	13.3	0.636	<0.001	<0.001
	Yes	45.1	13.3		52.5	16.5		<0.001	48.8	14.0		<0.001	50.5	14.6		<0.001	50.6	12.9		<0.001	<0.001
Phe	No	53.4	9.9	<0.001	60.7	10.7	0.003	<0.001	57.1	10.4	<0.001	<0.001	58.2	10.7	<0.001	<0.001	60.4	10.8	<0.001	<0.001	<0.001
	Yes	58.0	9.7		64.5	15.9		<0.001	60.7	10.4		<0.001	61.5	11.6		<0.001	63.1	11.9		<0.001	<0.001
Pro	No	114.9	48.0	<0.001	140.6	59.5	0.274	<0.001	125.5	52.1	<0.001	<0.001	124.1	50.8	<0.001	<0.001	144.7	52.3	0.017	<0.001	<0.001
	Yes	132.6	51.7		145.2	67.0		ns	144.1	49.9		<0.001	138.6	48.3		ns	153.7	53.4		<0.001	<0.001
Ser	No	115.6	27.4	<0.001	112.7	25.6	0.005	ns	109.8	25.9	<0.001	<0.001	109.5	26.7	<0.001	<0.001	107.6	26.7	<0.001	<0.001	<0.001
	Yes	103.3	24.5		102.6	42.4		ns	100.6	22.7		ns	102.1	21.9		ns	100.8	22.6		ns	0.223
Thr	No	120.7	33.7	0.484	123.1	37.8	0.473	0.036	119.3	32.9	0.017	ns	120.6	34.2	0.026	ns	124.6	35.4	0.501	<0.001	<0.001
	Yes	119.3	33.9		122.8	32.9		ns	122.6	34.4		ns	123.2	39.0		ns	126.9	32.1		0.005	0.023
Trp	No	51.0	11.6	<0.001	53.2	13.7	0.159	<0.001	53.6	11.9	<0.001	<0.001	53.2	11.9	<0.001	<0.001	56.5	11.9	0.019	<0.001	<0.001
	Yes	55.5	11.7		55.6	11.1		ns	57.5	12.9		0.005	55.7	12.6		ns	57.9	13.8		0.003	0.001
Tyr	No	57.0	14.7	<0.001	67.3	18.0	0.186	<0.001	62.6	14.8	<0.001	<0.001	64.7	16.7	<0.001	<0.001	68.3	16.3	<0.001	<0.001	<0.001
	Yes	61.5	16.1		69.4	22.3		0.003	67.0	16.1		0.000	68.0	17.9		<0.001	73.1	17.3		<0.001	<0.001
Val	No	188.4	49.2	<0.001	227.7	53.5	0.008	<0.001	209.4	54.6	<0.001	<0.001	208.4	54.5	<0.001	<0.001	233.3	53.1	<0.001	<0.001	<0.001
	Yes	224.4	50.0		242.0	66.0		0.001	237.3	48.5		<0.001	227.8	50.4		ns	244.3	48.0		<0.001	<0.001

p1 indicates the p-values for the two-tailed Mann-Whitney U-test for 2-independent samples (shown under each group of subjects); p2 indicates the p-values for the two-tailed Mann-Whitney U-test for 2-independent samples with adjustments by Bonferroni corrections for multiple comparisons with the corresponding values of no-LSRD group (shown under DM, dyslipidemia, hypertension and MetS); and p3 indicates the p-values for the two-tailed Kruskal-Wallis test for k-independent samples (shown in the last column). ns, not significant after adjustment for multiple comparisons.

In general, the concentrations of amino acids differed significantly when compared between the no-LSRD group and the LSRD groups (Kruskal-Wallis test, p < 0.05 to 0.001) (Table 2). Compared to the no-LSRD group, most of the amino acids showed an increase in concentration whereas Gly and Ser showed a decreasing trend in the latter groups. Overall, the significant differences between the no-LSRD group and each of the other four LSRD groups persisted after adjustments for multiple comparisons, particularly for the BCAAs and AAAs, and also for Ala, arginine (Arg), citrulline (Cit), Lys and ornithine (Orn) (Bonferroni-corrected Mann-Whitney U-test, p < 0.05 to 0.001).

### Association between PFAAs and HU in LSRDs

To confirm the association between PFAAs and elevated levels of UA in LSRDs, we divided the subjects according to the presence or absence of HU in the no-LSRD and LSRD groups and conducted binary (HU: no versus yes) logistic regression analyses evaluating the association between HU and each individual amino acid after adjusting for the variables relevant to each group of subjects. The odds ratios (ORs) derived from the mentioned logistic regression analyses are presented in Table 3. As the results of this study show, several amino acids showed consistent relationships with HU in both the no-LSRD group and the four LSRD groups.

**Table 3:** Logistic regression analysis for the association between hyperuricemia (HU) and individual amino acids for different groups of subjects without lifestyle-related diseases (no-LSRD) and with diabetes mellitus (DM), dyslipidemia, hypertension and metabolic syndrome (MetS) with adjustment for relevant potential confounding demographic and clinical factors: no-LSRD for age, gender, BMI, SBP, DBP, FPG, HbA1c, LDL-C, HDL-C and TG; DM for age, gender, BMI, SBP, DBP, LDL-C, HDL-C, TG and medication; dyslipidemia for age, gender, BMI, SBP, DBP, FPG, HbA1c and medication; hypertension for age, gender, BMI, FPG, HbA1c, LDL-C, HDL-C, TG and medication; and MetS for age, gender, BMI and medication. The odds ratios (OR) with 95% confidence intervals (CI) were estimated per IQR change in the concentrations of corresponding amino acids.

Aminoacid	No-LSRD				DM				Dyslipidemia				Hypertension				MetS			
	(HU: no, 2613; yes, 192)				(HU: no, 362; yes, 53)				(HU: no, 2655; yes, 552)				(HU: no, 2261; yes, 475)				(HU: no, 509; yes, 208)			
	OR	95% CI		p-value	OR	95% CI		p-value	OR	95% CI		p-value	OR	95% CI		p-value	OR	95% CI		p-value
		Lower	Upper			Lower	Upper			Lower	Upper			Lower	Upper			Lower	Upper	
Ala	0.91	0.72	1.15	0.417	1.20	0.76	1.91	0.430	1.28	1.09	1.51	0.003	1.07	0.90	1.27	0.430	0.90	0.71	1.13	0.374
Arg	0.76	0.60	0.96	0.024	0.93	0.60	1.43	0.739	0.93	0.81	1.07	0.304	1.02	0.88	1.18	0.816	1.06	0.84	1.32	0.642
Asn	0.73	0.58	0.91	0.007	0.94	0.63	1.40	0.745	0.94	0.82	1.08	0.364	1.02	0.88	1.18	0.805	1.11	0.89	1.39	0.348
Cit	1.32	1.05	1.65	0.016	1.37	0.98	1.92	0.068	1.16	1.01	1.33	0.036	1.24	1.07	1.42	0.003	1.34	1.09	1.64	0.005
Gln	0.66	0.52	0.83	<0.001	0.68	0.45	1.03	0.069	0.78	0.68	0.89	<0.001	0.88	0.76	1.02	0.099	0.99	0.80	1.23	0.945
Gly	0.71	0.56	0.90	0.004	0.79	0.52	1.19	0.253	0.73	0.63	0.84	<0.001	0.88	0.76	1.01	0.076	0.84	0.70	1.01	0.071
His	0.97	0.79	1.20	0.808	1.37	0.94	2.00	0.100	1.12	0.99	1.26	0.071	1.21	1.04	1.40	0.012	1.10	0.94	1.29	0.238
Ile	1.45	1.16	1.81	0.001	1.36	0.86	2.16	0.187	1.63	1.36	1.94	<0.001	1.46	1.22	1.75	<0.001	1.40	1.08	1.82	0.012
Leu	1.74	1.33	2.26	<0.001	1.97	1.19	3.24	0.008	1.82	1.50	2.21	<0.001	1.53	1.25	1.88	<0.001	1.48	1.13	1.93	0.004
Lys	0.99	0.78	1.26	0.934	1.56	1.04	2.35	0.031	1.12	0.98	1.28	0.097	1.20	1.03	1.40	0.017	1.25	1.00	1.55	0.046
Met	0.82	0.64	1.04	0.099	1.01	0.66	1.53	0.971	1.04	0.89	1.21	0.644	1.08	0.92	1.27	0.330	1.16	0.93	1.45	0.183
Orn	1.07	0.86	1.33	0.544	1.12	0.77	1.64	0.549	1.11	0.99	1.25	0.075	1.13	1.00	1.28	0.047	1.21	0.99	1.46	0.059
Phe	1.27	1.03	1.56	0.026	1.82	1.24	2.66	0.002	1.34	1.18	1.53	<0.001	1.45	1.26	1.67	<0.001	1.68	1.34	2.10	<0.001
Pro	0.89	0.73	1.09	0.271	1.20	0.78	1.85	0.404	1.18	1.04	1.34	0.010	1.09	0.94	1.26	0.265	1.22	1.00	1.49	0.047
Ser	0.51	0.39	0.66	<0.001	0.57	0.37	0.88	0.011	0.57	0.49	0.66	0.000	0.70	0.60	0.83	<0.001	0.68	0.54	0.86	0.001
Thr	0.74	0.59	0.93	0.011	0.93	0.62	1.41	0.745	1.03	0.91	1.16	0.670	1.03	0.90	1.19	0.667	1.16	0.96	1.39	0.118
Trp	0.88	0.70	1.10	0.251	1.35	0.87	2.10	0.174	1.14	0.99	1.31	0.076	1.01	0.88	1.17	0.847	1.19	0.95	1.48	0.133
Tyr	1.21	0.96	1.52	0.102	1.02	0.69	1.52	0.915	1.18	1.02	1.36	0.023	1.16	0.99	1.35	0.059	1.33	1.06	1.67	0.012
Val	1.66	1.28	2.15	<0.001	1.51	0.92	2.46	0.100	1.69	1.42	2.02	<0.001	1.38	1.14	1.66	0.001	1.24	0.96	1.59	0.099

Overall, all BCAAs (Ile, Leu, and Val) showed significant positive associations with HU [OR between 1.38 and 1.97; 95% confidence interval (CI) between 1.08 and 1.50 (lower) and 1.66 and 3.24 (upper), p < 0.05 to 0.001]; the exceptions were Ile in DM, and Val in DM and MetS. Cit (except in DM) and Phe also exhibited a significant positive associations with HU [OR between 1.16 and 1.82; 95% CI between 1.01 and 1.34 (lower) and 1.33 and 2.66 (upper), p < 0.05 to 0.001]. Moreover, such positive associations with HU were shown for Lys in DM, hypertension and MetS [OR between 1.20 and 1.56; 95% CI between 1.00 and 1.04 (lower) and 1.40 to 2.35 (upper), p < 0.05], and for Tyr in dyslipidemia and MetS [OR between 1.18 and 1.33; 95% CI between 1.02 and 1.06 (lower) and 1.36 and 1.67 (upper), p < 0.05]. Conversely, Ala and Pro in dyslipidemia [OR between 1.18 and 1.28; 95% CI between 1.04 and 1.09 (lower) and 1.34 and 1.51 (upper), p < 0.05 to 0.005] and His and Orn in hypertension [OR 1.13 and 1.21; 95% CI 1.00 and 1.04 (lower) and 1.28 and 1.40 (upper); p < 0.05] demonstrated individual positive associations with HU.

As shown in Table 3, Ser demonstrated significant inverse associations with HU for all groups of subjects [OR between 0.51 and 0.70; 95% CI between 0.37 and 0.60 (lower) and 0.66 and 0.88 (upper), p < 0.05 to 0.001] as did Gln and Gly for no-LSRD and dyslipidemia [OR between 0.66 and 0.78; 95% CI between 0.52 and 0.68 (lower) and 0.83 and 0.90 (upper), p < 0.005 to 0.001]. Conversely, Arg, asparagine (Asn) and threonine (Thr) showed significant inverse associations with HU in the no-LSRD group only [OR between 0.73 and 0.76; 95% CI between 0.58 and 0.60 (lower) and 0.91 and 0.96 (upper), p < 0.05 to 0.01].

## Discussion

In humans, individual amino acids play different metabolic or biochemical roles and make independent contributions to increasing LSRD risks^4,25,26^. Furthermore, each LSRD has a different relationship with amino acid metabolism^4,26^. Conversely, it is increasingly being emphasized that the changes in the concentration of blood UA levels might be associated with an increased risk of LSRDs^12,16–19^. However, despite the existence of close links between amino acids and the production of UA in the human body, the possible association between altered levels of amino acids and UA has not been investigated previously in LSRDs. To the best of our knowledge, this is the first study reporting on the association between altered levels of PFAAs and HU in DM, dyslipidemia, hypertension and MetS.

A significant age difference observed between the subjects without and with LSRDs in our study might have been caused by the fact that the prevalence of such chronic health conditions progressively increases with age and LSRDs are common amongst older adults. Our study showed that the prevalence of HU amongst men was much higher than that amongst women in all groups of subjects, which agrees with the existing literature^27,28^. The presence of higher levels of estrogen in women promoting the excretion of UA might be responsible for such a difference in UA levels between men and women^29^.

In this study, the results demonstrated that compared to the subjects without HU, subjects with HU had altered PFAA levels in all groups. In this study, such changes in PFAAs were less significant in the DM group which might have occurred because those patients with and without HU showed similar glycemic control: their FPG levels did not differ significantly. Another reason for this observation may be the relatively small number of subjects found eligible for this group. However, considering the trends of changes in PFAA concentrations between subjects with and without HU, it can be postulated that the state of HU is accompanied by an elevation in several amino acid concentrations and a depression in others. The lower concentrations of amino acids particularly for Gly and Ser that were observed in all groups of subjects with HU in our study is likely because they play important roles in the biosynthesis of purine and donate either amide nitrogen or carbon or both to the purine ring, which is utilized in increasing amounts for the formation of UA^30^. Moreover, our results demonstrated significant group differences for the levels of PFAAs between the no-LSRD and LSRD groups. However, as such differences disappeared after adjustments for multiple comparisons for a number of amino acids among the patients with HU, this might be explained by the fact that HU caused remarkable alterations in PFAA levels in both the no-LSRD group and other four LSRD groups.

As observed, the plasma concentrations of BCAAs and AAAs were found to be elevated in cases of LSRDs in this study. Our findings are consistent with the results from other research. For example, Wang *et al*. (2011) observed that a cluster of BCAAs (Ile, Leu, Val) and AAAs (Phe, Tyr) predicted the future development of type 2 diabetes up to 12 years prior to its onset^6^. McCormack *et al*. also described significant relationships of elevated concentrations of BCAAs with future insulin resistance amongst children and adolescents^2^. Increased levels of circulating BCAAs may promote insulin resistance possibly via the disruption of insulin signalling in skeletal muscles through activation of the mTOR, JUN and IRS1 signalling pathways^6^. Conversely, elevations in the plasma concentrations of AAAs (particularly Phe and Tyr) might have resulted from the repression of tyrosine aminotransferase under an insulin-resistant state^4,31^. In the blood, approximately 40% of the free essential amino acids are comprised of BCAAs^32^. Increased levels of AAAs (Phe and Tyr) may have been driven by higher levels of BCAAs in the blood because BCAAs and AAAs compete for transport into mammalian cells by the common large neutral amino acid transporter (LAT1)^33^. Furthermore, compared to the no-LSRD subjects, we observed increased levels of other amino acids in DM such as Ala, Arg, Gln, His and Met, and a decreased level of Gly. Increased levels of gluconeogenesis-related amino acids, such as Ala and Gln, likely reflect impaired insulin sensitivity prior to the elevation of fasting glucose and insulin levels^4^. Conversely, the observed reduction in the level of Gly might have been caused by its enhanced utilization for accelerated gluconeogenesis by hepatocytes that are characteristic for the diabetic condition^34^. Furthermore, from the current findings, it seems possible that increased insulin levels may promote amino acid uptake into the skeleton muscles and inhibit protein breakdown, leading to reduced circulating amino acid concentrations^35^.

As our data show, the concentrations of several amino acids differed between the subjects without LSRDs and with hypertension, which agrees with the findings of a previous study^4^. The exact mechanisms underlying the association between amino acids and hypertension have not been clarified yet. It has been suggested that advanced glycation end products (AGEs) produced from the glycation of amino acids induce the expression of angiotensin II, which in turn increases blood pressure^36^. In our study, concentrations of Ala along with Met and Leu showed elevations amongst the subjects with hypertension. In the INTERMAP epidemiological study^37^, metabolic phenotyping demonstrated a strong association of urinary excretion of Ala with higher blood pressure amongst the participants. Furthermore, the transamination of Leu supports the formation of glutamate, the transamination of which generates Ala^38^. Moreover, Leu may act as an inhibitor of nitric oxide/NO synthesis by the endothelial cells^39^. Stühlinger *et al*.^40^ postulated that Met could cause an elevation in blood pressure because it is a precursor of homocysteine, which inhibits the production of endothelial NO by causing the accumulation of asymmetric dimethylarginine (ADMA), a competitive inhibitor of the latter.

In this study, compared to the no-LSRD group, UA levels were significantly higher amongst four groups of subjects with LSRDs. In a previous study from Japan, elevation in the concentration of serum UA was found to increase the risk of type 2 diabetes in a 6-year follow-up study conducted amongst 2310 Japanese male workers aged 35–59 years^41^. Insulin requires NO to stimulate glucose uptake by skeletal muscles. However, the bioavailability of endothelial NO is suppressed in HU, which may play a key role in the development of insulin resistance and/or hyperinsulinemia^16,42^. Hyperinsulinemia may also cause increased reabsorption of UA in the kidneys and further increase in the level of plasma UA^19^.

Existing evidence also suggests a clear relationship of HU with hypertension. HU causes endothelial dysfunction and impaired NO production and promotes vascular smooth muscle cell proliferation and arterial stiffening^20,43^. Conversely, the restoration of endothelium-dependent vasodilation and raised circulating antioxidant defenses by administering UA has been reported in the literature^44^. However, such beneficial effects of UA were observed after the acute administration of it, and the effects of chronic exposure to elevated serum UA concentrations might differ from the former effects. By contrast, published studies reported that HU is associated with an increased generation of free radicals and oxidative stress, platelet adhesion and aggregation^21,43^. Furthermore, HU causes activation of the renin–angiotensin system with an increase in sodium resorption^45^. All these lead to the development of hypertension with the subsequent development of increased renal vascular resistance and reduced renal blood flow, which in turn may decrease the renal urate secretion in the proximal tubule and thus causing further increases in the level of plasma UA^43,46,47^.

**Figure 3:**
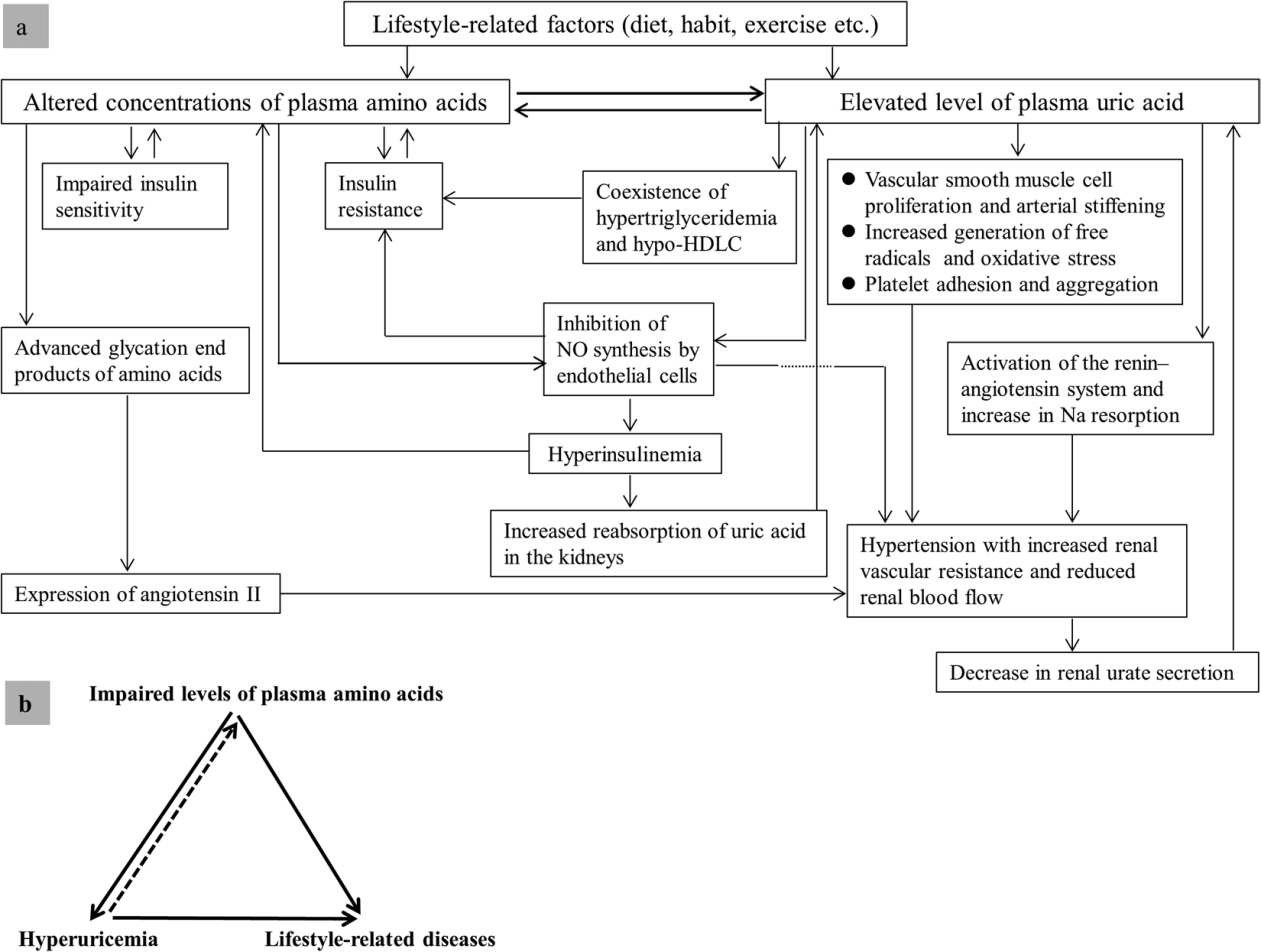
Hypothetical schematic diagram of probable causes and consequences of altered plasma levels of amino acids and uric acid (panel a), and triad for probable amino-uric interaction in the development of lifestyle-related diseases (panel b).

As observed in the present study, subjects with HU and with LSRDs had significantly higher TG and LDLC levels, and significantly lower HDLC levels. These findings agree with the existing literature in that hyperuricemic men and women have displayed the coexistence of hypertriglyceridemia and hypo-HDLC^28,48,49^. In a previous study, the association between low HDLC and elevated levels of TG has been suggested to be linked with the insulin resistance syndrome^49^. Therefore, we postulate that the coexistence of hypo-HDLC and hypertriglyceridemia in HU may play an important role in the development of LSRDs. Conversely, another previous study has shown the positive association of serum UA with MetS^28^. UA may be a cause of the MetS, possibly due to its ability to inhibit endothelial function^17^. The above-discussed causes and consequences of altered levels of PFAAs and plasma UA and their roles in the development of LSRDs are summarized in Fig. 3a.

In the present study, we first investigated the correlations of PFAAs with UA including all subjects with or without HU and with no adjustments for any potential confounders. Our results revealed that most PFAAs were positively correlated with UA, and Gly and Ser were negatively correlated with UA. Our findings cannot be compared to those of others due to the absence of such information in the published literature. However, our findings suggest the possibility of a close relationship between amino acids and UA in human blood.

Next, we confirmed the association between PFAAs and the presence of HU in LSRDs. In our study, the associations between PFAAs and HU revealed by logistic regression analysis after adjustments for potential relevant confounders produced different response patterns in a number of amino acids amongst subjects in the no-LSRD group and the LSRD groups. For example, the positive associations of Ala and Pro, and His and Orn with HU were characteristic only for dyslipidemia and hypertension, respectively. Intriguingly, although several PFAAs (Arg, Asn and Thr) had significant inverse associations with HU in no-LSRD subjects, such significant relationships were not observed in LSRD subjects. However, the exact mechanisms underlying the observed association between the PFAA levels and HU are complex and cannot be explained based on the current study findings. It was also not possible to discern whether the change in the PFAA levels was a cause or merely a consequence of the changes in the UA levels, and vice versa.

There is a close link between diet, amino acids and blood UA level. It has been postulated that the UA level in the blood is determined by a balance amongst dietary purine intake, hepatic purine production and urinary UA excretion^20^. Magnusson *et al*.^50^ mentioned that an obvious factor that can alter amino acid levels in plasma is diet. For essential amino acids, such changes could be even greater^51^. However, along with carbohydrates and saturated fat, a diet rich in purines may lead to HU^21^. In a study conducted by Choi *et al*.^52^ including both men and women, serum UA levels increased significantly with an increasing consumption of purine-rich foods (meat and seafood), and decreased significantly with the consumption of foods low in purine content (dairy products). However, in the current study, we did not collect detailed dietary information from the study participants and diet was also not considered as a covariate in the logistic regression analysis. Therefore, our study results should be interpreted with caution because the possibility of biased results due to the lack of information on dietary habits cannot be excluded. However, we assume that any such effects on the current study findings were somewhat limited by the fact that we included a control group of subjects without LSRDs and the results were compared between the no-LSRD and LSRD groups after adjustment for several potential confounders. Furthermore, the association between purine consumption and UA has been reported to be independent of total protein intake and other factors such as age, sex, BMI, serum creatinine level, hypertension, alcohol use, and diuretic use^52^.

Based on our findings of the similarities and dissimilarities in PFAA concentrations amongst subjects with and without LSRDs and the findings from previously published studies considered together, it can be postulated that a close link exists between PFAA**s** and plasma UA. We hypothesize that altered PFAA levels triggered by lifestyle-related factors possibly induce changes in the plasma level of UA, causing an increase in the latter. Furthermore, it may be possible that such an altered UA level promotes further changes in the PFAA levels, thus causing a vicious circle between PFAAs and UA (Fig. 3b). Each of the mentioned LSRDs might be a consequence of the specific interaction between altered amino acid metabolism and UA (mentioned as the amino-uric interaction in Fig. 3b). However, the exact role of PFAAs and UA in LSRD remains to be properly established in future longitudinal studies.

### Limitations

Interpretation of the current study findings should be considered in light of several potential limitations. First, background information on physical activity smoking status and alcohol consumption were not assessed in this study, which might have biased the results. However, we assume that the effects of these factors on the study findings were minimal due to the reasons mentioned previously. Second, we did not collect data on the medications such as those blocking UA production (xanthine oxidase inhibitors) or the use of hormone replacement therapy amongst female participants. However, we firmly believe that this does not influence the outcome of our study findings because the use of such drugs lowering the blood UA level and/or promoting the excretion of UA would mean an underestimation of the current results. Third, the generalization of the study findings is somewhat limited by the fact that this study was conducted amongst the Japanese population, and the age of the study subjects significantly differed between the groups with and without LSRDs. Therefore, the current findings should be validated in future cohort studies including large and diverse populations amongst age-matched groups. Fourth, the cross-sectional nature of the current study design does not allow us to speculate any causality or temporality of the associations observed in this study. Therefore, caution is required when interpreting the information including the direction of the arrows shown in Fig. 3 of this study as the figure is merely a schematic representation of the hypothetical relationships between altered levels of PFAAs and UA that are based on the existing published literature and the relevant discussion on the current study findings. However, as we believe, our findings have generated interesting and important findings and the relevant hypotheses must be tested and clarified in future longitudinal studies.

### Conclusions

For the first time, our study provides evidence for a strong association between the plasma levels of amino acids and HU in DM, dyslipidemia, hypertension, and MetS after adjustment for possible confounding factors. The current findings might help increase our understanding of the role of alterations in PFAA levels and accompanying HU in the development of LSRDs. Our data considered collectively with the findings from the relevant existing literature, also suggest the possibility of interplay between PFAAs and UA in the pathophysiology of LSRDs. In the future, longitudinal studies should target individuals without LSRDs and measure any changes in UA or PFAAs and confirm which occurs earlier prior to the development of LSRDs. Furthermore, it would be necessary to investigate the effects of dietary supplementation of selective amino acids according to individual requirements on metabolic controls and plasma UA levels amongst patient populations, which would be extremely valuable in establishing the strategies for the prevention and early detection, monitoring and management of LSRDs.

## Methods

### Subjects

A total of 8589 subjects who underwent their annual health check-up during 2009 to 2011 in Shimane Prefecture, Japan and for whom workplace health examination was not applicable were recruited for this study. The health examinations comprised physical examinations, clinical and laboratory tests, and a self-administered questionnaire that included personal and medical history. Among the subjects, 1278 were excluded from the analyses because data on UA and/or PFAAs were not available. Furthermore, 1 subject was excluded due to a lack of demographic data, and another subject was excluded due to abnormally high UA data. Finally, 7309 subjects were included in the current study. The subjects had no serious health problems such as cancer or renal failure. The schematic of the workflow used in the present study is depicted in Fig. 1.

### Ethical issues

The current study was conducted in accordance with the Declaration of Helsinki. An oral explanation of the study protocol was made in detail to the study participants and written informed consent to participate in this study was obtained from all of them. The protocol of the present study was approved by the relevant institutional review board of Shimane University (20100129-3) and Yamaguchi University (H25-26-2).

### Measurement of laboratory variables and PFAAs

Blood samples were obtained from the subjects after an 8-hour fast. Serum levels of HDLC, LDLC, and TG were determined enzymatically^3^. For the measurements of FPG and HbA1c, the hexokinase method and the latex agglutination immunoassay were used, respectively. Plasma UA levels were measured using the uricase-HMMPS method by L-type UA.M kit (Wako Pure Chemical Industries, Ltd., Japan).

Blood samples were collected and analysed for PFAA concentrations following the protocol previously described elsewhere^4,26,53,54^. Briefly, after overnight fasting, five ml of venous blood samples were collected from the cubital vein of the seated subjects into tubes containing ethylenediaminetetraacetic acid (EDTA; Termo, Tokyo, Japan). The tubes were placed on ice immediately and stored there for approximately 15 min. After centrifugation of the tubes at 4 °C and 3,000 rpm for 15 min, the plasma was immediately separated into tubes and stored at −80 °C. The tubes were stored there until (within 2 weeks to 2 months) the desired analysis for PFAAs. The PFAA concentrations were measured by high-performance liquid chromatography–electrospray ionization–mass spectrometry (HPLC–ESI–MS) followed by precolumn derivatization which allows such measurements with high accuracy. In this study, the absolute concentrations (in μmol/L) of the following 19 amino acids were measured: Ala, Arg, Asn, Cit, Gln, Gly, His, Ile, Leu, Lys, Met, Orn, Phe, Pro, Ser, Thr, Trp, Tyr, and Val. The measurements of other genetically encoded amino acids such as glutamate, aspartate, and cysteine were not performed due to their instability in the blood^3,4^.

### Clinical assessments

In this study, DM was defined as an FPG level of ≥126 mg/dL, a HbA1c level of ≥6.5%, and/or the use of medication for DM. Dyslipidemia was defined in individuals as an LDLC level of ≥140 mg/dL, an HDLC level of <40 mg/dL, a TG  level of ≥150 mg/dL, and/or the use of medication for dyslipidemia. Hypertension was defined as an SBP of ≥140 mmHg or a DBP of ≥90 mmHg and/or the use of antihypertensive medications. MetS was defined according to the following Japanese criteria used for diagnosis of the syndrome^3,4^: visceral obesity (waist circumference ≥ 85 cm in males and ≥90 cm in females) plus at least 2 of the following three components:

1. HDLC < 40 mg/dL, TG ≥ 150 mg/dL, and/or the use of medication for dyslipidemia;
2. FPG ≥ 110 mg/dL and/or the use of medication for DM; and
3. blood pressure ≥ 130/85 mmHg and/or the use of antihypertensive medication.

Based on the available literature, we defined HU as a plasma UA level of ≥7 mg/dL in men and ≥6.0 mg/dL in women^9,48,55,56^.

### Statistical analyses

In this study, the continuous variables were expressed as the median and IQR. The differences between the groups for demographic and clinical variables were examined by the Mann-Whitney U-test for 2-independent samples and the Kruskal-Wallis test for k-independent samples. For multiple comparisons with the no-LSRD group, the adjustments were made by Bonferroni corrections as necessary. For the categorical variables, the differences between the groups were assessed with the Chi-square (χ^2^) test. Spearman’s rank correlation analysis was performed between individual PFAA and UA concentrations in the no-LSRD group and the LSRDs groups. The association between HU and individual amino acids in each study sample was investigated by logistic regression analyses. For this purpose, all of the amino acids were scaled to multiples of 1 IQR and corresponding odds ratios (OR), 95% CI and p-values were obtained. To exclude the crossover effects amongst diseases on the single PFAA level in all groups of patients, the logistic regression analysis was performed with adjustments for the potential confounding factors showing significant group differences except for the relevant factors used in the diagnosis of the four groups of LSRDs separately, in line with a previously published study^3^: DM for age, gender, BMI, use of medication (yes, no), SBP, DBP, LDL-C, HDL-C and TG; dyslipidemia for age, gender, BMI, use of medication, SBP, DBP, FPG and HbA1c; hypertension for age, gender, BMI, use of medication, FPG, HbA1c, LDL-C, HDL-C and TG; and MetS for age, gender, BMI, and use of medication. The analyses with data on subjects without LSRDs (no-LSRD) were performed with adjustments for the following possible confounding variables: age, gender, BMI, SBP, DBP, FPG, HbA1c, LDL-C, HDL-C and TG. The data used in this study were analyzed anonymously. All statistical tests were considered two-tailed, and a value of p < 0.05 was set as the significance level^4^. The software package SPSS version 22 for Windows (SPSS Inc., Chicago, IL, USA) was used to perform the statistical analyses except for the Spearman’s rank correlations which were calculated and plotted using the package ‘corrplot’ in the R statistical software v.3.4.0^57^.

### Availability of data and materials

Requests for data and materials should be addressed to the corresponding author.
